# Quantification of the natural history of visceral leishmaniasis and consequences for control

**DOI:** 10.1186/s13071-015-1136-3

**Published:** 2015-10-22

**Authors:** Lloyd A C Chapman, Louise Dyson, Orin Courtenay, Rajib Chowdhury, Caryn Bern, Graham F. Medley, T. Deirdre Hollingsworth

**Affiliations:** School of Life Sciences, University of Warwick, Gibbet Hill Campus, Coventry, CV4 7AL UK; Country Programme Manager - Bangladesh, KalaCORE Programme, Dhaka, Bangladesh; Department of Medical Entomology, National Institute of Preventive and Social Medicine (NIPSOM), Mohakhali, Dhaka, Bangladesh; UCSF School of Medicine, 550 16th Street, San Francisco, CA 94158 USA; London School of Hygiene and Tropical Medicine, Keppel Street, London, WC1E 7HT UK

**Keywords:** Visceral leishmaniasis, Natural history, Control, Diagnostics, Multi-state Markov model, Indian sub-continent

## Abstract

**Background:**

Visceral leishmaniasis has been targeted for elimination as a public health problem (less than 1 case per 10,000 people per year) in the Indian sub-continent by 2017. However, there is still a high degree of uncertainty about the natural history of the disease, in particular about the duration of asymptomatic infection and the proportion of asymptomatically infected individuals that develop clinical visceral leishmaniasis. Quantifying these aspects of the disease is key for guiding efforts to eliminate visceral leishmaniasis and maintaining elimination once it is reached.

**Methods:**

Data from a detailed epidemiological study in Bangladesh in 2002–2004 was analysed to estimate key epidemiological parameters. The role of diagnostics in determining the probability and rate of progression to clinical disease was estimated by fitting Cox proportional hazards models. A multi-state Markov model of the natural history of visceral leishmaniasis was fitted to the data to estimate the asymptomatic infection period and the proportion of asymptomatic individuals going on to develop clinical symptoms.

**Results:**

At the time of the study, individuals were taking several months to be diagnosed with visceral leishmaniasis, leading to many opportunities for ongoing transmission. The probability of progression to clinical disease was strongly associated with initial seropositivity and even more strongly with seroconversion, with most clinical symptoms developing within a year. The estimated average durations of asymptomatic infection and symptomatic infection for our model of the natural history are 147 days (95 % CI 130–166) and 140 days (95 % CI 123–160), respectively, and are significantly longer than previously reported estimates. We estimate from the data that 14.7 % (95 % CI 12.6-20.0 %) of asymptomatic individuals develop clinical symptoms—a greater proportion than previously estimated.

**Conclusions:**

Extended periods of asymptomatic infection could be important for visceral leishmaniasis transmission, but this depends critically on the relative infectivity of asymptomatic and symptomatic individuals to sandflies. These estimates could be informed by similar analysis of other datasets. Our results highlight the importance of reducing times from onset of symptoms to diagnosis and treatment to reduce opportunities for transmission.

**Electronic supplementary material:**

The online version of this article (doi:10.1186/s13071-015-1136-3) contains supplementary material, which is available to authorized users.

## Background

Visceral leishmaniasis (VL) in the Indian sub-continent (ISC) is a disease caused by chronic infection with the protozoan parasite *Leishmania donovani*, transmitted by the *Phlebotomus argentipes* sandfly. Clinically manifest visceral leishmaniasis, also called kala-azar (KA), is progressive with a high mortality rate, and characterized by prolonged fever and an enlarged liver and/or spleen. Clinical and laboratory diagnostics are imprecise [1–4], partly because only a small proportion of infected individuals develop disease (so that the presence of infection alone is not diagnostic), and partly because the clinical features of VL overlap with those of other endemic diseases (e.g. hyperreactive malarial splenomegaly, typhoid fever and disseminated tuberculosis), so that clinical presentation alone is not diagnostic. The current diagnosis generally relies on clinical features, specifically a fever lasting at least 14 days and a palpable liver/spleen (hepatomegaly/splenomegaly), and elevated rK39 antibodies (based on immuno-chromatographic assay) rather than evidence of active current infection. This combination of the duration of infection and rK39 rapid testing has high sensitivity [5].

Epidemiologically, KA is spatially highly heterogeneous with focal ‘hotspots’ of infection that move over time, and periodic epidemics on a timescale of decades [6, 7]. The control campaign in the ISC, which has been running since 2005, has focused on elimination as a public health problem (less than 1 new case per 10,000 people per year), defined at local geographical scales (per subdistrict in both India and Bangladesh, known as an upazila in Bangladesh). Progress has been made towards the target by implementing novel case detection strategies, rapid diagnostic testing and vector control activities. In Bangladesh, KA incidence declined in all districts in the 8 years following the start of the control campaign (2006–2013) from the previous 8 years (1998–2005) [8]. The highest number of cases was reported in 2006, after which the annual number of cases decreased significantly. In the period from 2008 to 2013, only 16 upazilas had average incidence rates above the elimination target (ranging from 1.06-18.25/10,000 people/year) [8]. In Nepal, where KA was only endemic in south-eastern districts neighbouring the state of Bihar in India and incidence rates were much lower (1-10/10,000 people/year in 2007–2008 [9]), the elimination target has been reached for two consecutive years. However, Bihar, which accounts for 70-80 % of the KA cases in India [10], is still far from the target with an estimated incidence of 22–29.8/10,000 people/year in 2006–2007 [11], and more recent estimates of 1–5 cases/10,000 people/year [10, 12].

Although vector control activities (in particular indoor residual spraying (IRS)) are a pillar of the elimination programme [13, 14], they appear to have uncertain and variable effectiveness, likely due to sub-optimal implementation and, in some areas, insecticide resistance [15–17]. However, a randomised control trial of IRS and insecticide-treated bed nets in Bangladesh from 2006 to 2007 showed a 70-80 % reduction in sandfly density up to 5 months post intervention [18], and recent modelling of IRS suggests that in low and medium endemicity settings (5–10 KA cases/10,000 people/year) effective IRS may be sufficient to reach the 1 case/10,000 people/year elimination target [19].

Progress in KA case reduction over the past decade has been largely attributed to improved timeliness of diagnoses and more effective treatment [20]. Given that the 'natural' epidemiology of the disease is typified by recurrent epidemics followed by long periods of low incidence, and noting that the current control is dependent on substantial external resources, effort and clinical awareness, there is considerable potential for future resurgence without a sustained elimination effort [20, 21]. Other major issues for the elimination programme include high levels of under-reporting (the ratio of actual to reported KA cases in the ISC is estimated to range between 2:1 and 8:1 [22, 23]), and the unknown contribution of asymptomatically infected individuals, who potentially form a large infectious reservoir, to transmission [24, 25].

Mathematical and statistical modelling of infectious diseases has a successful history of combining epidemiological data, biological understanding and clinical knowledge into quantitative frameworks that can be used to both interpret disease incidence (in terms of infection patterns) and predict the impact of proposed interventions. Visceral leishmaniasis is unusual in that there have been relatively few previous modelling attempts, mostly driven by the lack of quantitative data [26]. There are, to our knowledge, only two recent, high quality, longitudinal epidemiological studies: the KALANET bed net trial in India and Nepal between 2006 and 2009 [27, 28], and the studies of Bern et al. in Bangladesh from 2002 to 2010 [29–31]. Consequently, there is still a large amount of uncertainty about the natural history of the disease, in particular about its incubation period and the proportion of asymptomatically infected individuals that develop KA. From here on we treat the incubation period as being synonymous with the duration of asymptomatic infection, since in our modelling we do not initially distinguish between asymptomatically infected individuals that develop KA and those that do not, but we note that the duration of asymptomatic infection may be different for the two groups and also test this hypothesis (see Additional file 1). Previous estimates for the incubation period have ranged from 2 to 6 months [32–34], while estimates for the proportion of asymptomatic individuals that progress to KA have varied hugely, from 0.33 % [33] to 25 % [31]. Better quantification of these aspects of the disease is critical for developing effective models, guiding efforts to eliminate VL and maintaining elimination once it is reached.

Towards this end, we analyse the prospective, longitudinal data from a 3-year study in Bangladesh in the period 2002–2004 (details of the study and epidemiological analyses have been reported elsewhere [29, 31]). We use annual data on rK39 positivity and positivity of the leishmanin skin test (LST), together with KA diagnosis, to estimate the rates of progression between different disease states. The aim is to provide preliminary, quantitative estimates of waiting times (i.e. times spent in each state) and paths of progression that will feed into future transmission model development.

## Methods

### Data

The study took place in a single community in Fulbaria upazila, Mymensingh district, Bangladesh between January 2002 and June 2004. Fulbaria was chosen due to its high reported KA incidence for the three years prior to the study. In 2002 the community had a population of approximately 12,000 people and was divided into 9 'paras' (sections), of 100–500 houses each. Cross-sectional household surveys of the 3 paras with the highest reported KA incidence were conducted from January to April in 2002, 2003 and 2004. All individuals who lived at least 6 months in the study area in the 3 years before the first survey in 2002 were included. The protocol was approved by the International Centre for Diarrhoeal Disease Research, Bangladesh (ICDDR,B) Research and Ethical Review Committees and the Institutional Review Board of the Centers for Disease Control and Prevention (CDC).

The data recorded included demographic information (age and sex), present and past KA cases (back to 1999), and risk factors (such as sleeping location, bed net use, diet, and animal ownership). For participants ≥ 3 years of age, capillary blood samples were taken for serology testing and the leishmanin skin test (LST) was applied intradermally. The blood samples were tested by an enzyme-linked immunosorbent assay (ELISA) with recombinant K39 (rK39) antigen [35, 36] and a modified protocol that included a standard titration curve of a pool of known positive sera on each plate of blood samples [37]. Concentration units (CU) were assigned to the standard titration curve (with the highest concentration on the curve assigned a value of 1000CU), and the optical density of the serum specimens converted into CU. The positive ELISA cut-off was set at 20CU—the 99th percentile of the distribution of ELISA readings from 38 individuals living in a non-VL-endemic region of Bangladesh. A second cut-off of 61CU was introduced for diagnosis of active KA, with a sensitivity and specificity of 97 % and 98.9 % respectively for sera from the study population based on receiver-operator-characteristic analysis [37].

The antigen for the LST was a suspension of 5 × 10^6^ promastigotes/mL of the WHO-approved MHOM/TN/80/IPT1 strain of *L. infantum*. The test was applied following the standard protocol: 0.1 mL of antigen was injected intradermally on the inside of the forearm and 48–72 h later the induration of the skin measured in two perpendicular directions [38, 39]. In accordance with international consensus, the LST result was deemed positive if the mean of the two measurements was ≥ 5 mm [31, 40]. There was evidence of loss of leishmanin potency in this study in the 2003 and 2004 survey rounds [30], and so at later time points the number of individuals with positive LST reactivity is likely to be an underestimate (testing showed that the *L. infantum* antigen had a sensitivity of 70 % compared with *L. amazonensis* antigen in 2004).

A past case of KA was defined as an illness with ≥ 2 weeks of fever and at least one of: weight loss, abdominal swelling or skin darkening, with clinical improvement after 20 days of intramuscular injections of sodium stibogluconate (SSG) (the treatment for KA prescribed by national guidelines at the time). A present case of KA was defined as one that fulfilled the same definition plus splenomegaly and/or hepatomegaly and a positive rK39 ELISA result or rK39 dipstick test [29].

In total, data was collected on 2,410 out of 2507 individuals living in 509 houses in the 3 paras during the study. Of these individuals, 47 % were male and 53 % were female, and 2,152 had at least one rK39 ELISA or LST reading between 2002 and 2004. There were 182 cases of KA from the start of 1999 to the end of the study in June 2004: 125 cases with onset before 2002 and 57 with onset from 2002 to the end of the study (see Table 1). There were only 5 relapses to active KA following treatment, and the incidence of post kala-azar dermal leishmaniasis (PKDL) was very low, with only 4 confirmed cases out of the 182 KA cases (all of which were in 2004). Consequently, we have not included development of PKDL in our modelling.

**Table 1:** Summary of the data

Year	Reported number of KA cases	Reported number of KA deaths	Reported number of non-KA deaths	rK39 ELISA		LST	
				Positive	Negative	Positive	Negative
1999	17	0	4	-	-	-	-
2000	50	1	9	-	-	-	-
2001	58	6	9	-	-	-	-
2002	27	5	16	312 (19 %)	1301 (81 %)	530 (35 %)	1000 (65 %)
2003	24	2	14	284 (15 %)	1553 (85 %)	453 (26 %)	1294 (74 %)
2004 (to June)	6	2	0	252 (14 %)	1587 (86 %)	134 (19 %)	565 (81 %)

### Statistical analysis

Following the identification of delays between onset of symptoms and diagnosis and treatment [20, 41], a descriptive analysis of the key time periods in the data was performed. To investigate the impact of serological status on progression to disease, we analysed the risk of progression to KA for those with a particular sero-status at baseline, and those who seroconverted during the study. Kaplan-Meier curves were plotted and Cox proportional hazards regression models fitted to test for associations between (i) initial rK39 seropositivity and KA and (ii) rK39 seroconversion and KA, following a previous analysis by Hasker et al. [28].

Hasker et al. analysed serology data from four different cohorts in two large studies—two from the KALANET trial in India and Nepal, and two from the Tropical Medicine Research Centre (TMRC) project run in Bihar, India since 2008 [42, 43]—to determine the association between rK39 and direction agglutination test (DAT) antibody titres and progression to KA. For the KALANET trial, rK39 ELISA results were available for 2006 only, so only the risk of KA as a function of baseline seropositivity could be assessed, but for the TMRC surveys blood samples were tested using rK39 ELISA at each survey, so seroconversion was also investigated. Hasker et al. found that there was a strong association between high rK39 titres at baseline and progression to KA, and an even stronger association between seroconversion to a high titre and subsequent progression.

In our analysis, we take the 2002 serology survey as the baseline survey and the 2003 survey as the follow-up survey. We use the rK39 ELISA cut-offs described above to define seronegativity (rK39 ELISA reading < 20CU), moderate seropositivity (20CU ≤ rK39 ELISA < 61CU) and strong seropositivity (rK39 ELISA ≥ 61CU). These differ slightly from the cut-offs used in the Hasker study, in which the cut-off for seropositivity was given by the mean optical density of known negative sera plus two standard deviations, and the cut-off for strong seropositivity was determined by the percentage point optical density with the highest combined sensitivity and specificity for identifying individuals diagnosed with KA in the last 2 years. Since the cut-off for strong seropositivity for our data was determined using samples from individuals with active KA as positive controls, it is likely that it corresponds to a higher rK39 titre and is more specific for KA. Nevertheless, the cut-offs for the second TMRC cohort in [28], which was from a higher endemicity region, correspond closely to those used in our analysis.

For the analysis of KA progression risk with seroconversion, we classified seroconvertors from the 2002 survey to the 2003 survey into different groups. Individuals that sero-deconverted from being either strongly seropositive or moderately seropositive to seronegative were grouped together (sero-deconvertors), as were those that remained either seronegative or seropositive (non-convertors), who were taken as the reference group. Individuals whose titre increased between surveys were grouped into seroconvertors (who went from being seronegative to seropositive) and strong seroconvertors (who went from being seronegative or seropositive to strongly seropositive). People that were strongly seropositive at both surveys were treated as a separate group.

### Multi-state Markov model of natural history of VL

Multi-state Markov models provide an informative way of analysing the natural history of a disease, by describing how an individual moves through a series of disease states (e.g. healthy, infected, recovered, dead) in continuous time. The movement of individuals between states is governed by a set of transition intensities, *q*_*rs*_ (*r*, *s* = 1, …, *R*), each of which represents the instantaneous risk of moving from state *r* to state *s* for *r* ≠ *s* (${q}_{rr}:=-{\displaystyle \sum_{s\ne r}}{q}_{rs}$), where *R* is the number of states. The transition intensities may depend on time *t* and a set of (potentially individual-specific) explanatory variables *z* (i.e. *q*_*rs*_ = *q*_*rs*_(*t*, *z*)), and are summarised in an *R* × *R* matrix, *Q*, whose rows sum to zero. The aim of fitting the multi-state model to data on observations of individuals’ disease states is to estimate the transition intensity matrix *Q*.

Multi-state Markov models are particularly useful for modelling panel data on disease progression, such as that described above, where individuals are observed at approximately regular intervals, but the exact times between follow-up visits vary and limited information is available about the individuals between follow-up visits. This means that changes in individuals’ disease states generally occur at unknown times.

**Fig. 1:**
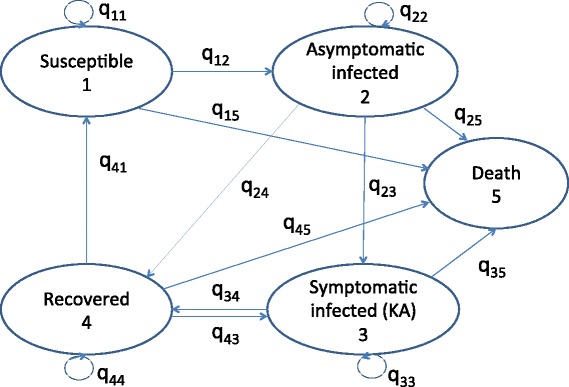
Flow diagram for multi-state Markov model of natural history of VL

Following Stauch et al. [33] and with a view to developing a transmission model, we model the natural history of VL as shown in Fig. 1. Individuals are classified into 5 different disease states—susceptible, asymptomatically infected, symptomatically infected (active KA), recovered/dormant, and dead—according to their KA status and the results of the rK39 ELISA and LST as shown in Table 2 (see Table A1 in Additional file 1 for the full classification including censored states for missing tests). Susceptible individuals (state 1) have negative rK39 ELISA and LST readings, are not currently symptomatic, and their most recent rK39 ELISA test was negative. If individuals have a positive ELISA, but a negative LST and have not previously suffered KA they are classed as being asymptomatically infected (state 2). On development of symptoms an individual is recorded as having KA (state 3) and remains in this state from the date of fever onset to the end of treatment or, if the individual is untreated, one year after the onset of symptoms (98 % of patients that have KA during the study have a date of onset of symptoms and 91 % have a date of start of treatment; prior to and after the study period these data are often missing and so these dates are recorded as unknown or uncertain). However, asymptomatically infected individuals may also progress to a 'recovered/dormant' state (state 4) without developing symptoms. Recovered/dormant individuals are classified as those who are LST positive, have recovered from their KA symptoms or have sero-deconverted from positive to negative rK39 ELISA. Recovered/dormant individuals may relapse to KA or return to being susceptible. Deaths due to KA and other causes (state 5) are included in the dataset, so individuals may be absorbed from any of states 1 to 4 into state 5.

**Table 2:** Classification of individuals into different disease states in multi-state model

Disease state	Description	rK39 ELISA	LST	KA status	Previous rK39 ELISA
1	Susceptible	−	−	−	−
2	Asymptomatic infected	+	−	−	+ / −
3	Symptomatic infected (KA)	+	−	+	+ / −
4	Recovered/dormant	−	−	−	+
		+ / −	+	−	+ / −
		+	−	− (post-KA)	+ / −
5	Dead	NA	NA	NA	NA

The transition intensity matrix for this 5-state model of VL is

$$ Q=\left(\begin{array}{ccccc}\hfill {q}_{11}\hfill & \hfill {q}_{12}\hfill & \hfill 0\hfill & \hfill 0\hfill & \hfill {q}_{15}\hfill \\ {}\hfill 0\hfill & \hfill {q}_{22}\hfill & \hfill {q}_{23}\hfill & \hfill {q}_{24}\hfill & \hfill {q}_{25}\hfill \\ {}\hfill 0\hfill & \hfill 0\hfill & \hfill {q}_{33}\hfill & \hfill {q}_{34}\hfill & \hfill {q}_{35}\hfill \\ {}\hfill {q}_{41}\hfill & \hfill 0\hfill & \hfill {q}_{43}\hfill & \hfill {q}_{44}\hfill & \hfill {q}_{45}\hfill \\ {}\hfill 0\hfill & \hfill 0\hfill & \hfill 0\hfill & \hfill 0\hfill & \hfill 0\hfill \end{array}\right). $$

We will assume that the *q*_*rs*_ are independent of the number of individuals in each state, and thus are time-independent. For this model, the proportion of those asymptomatically infected who develop symptoms (excluding individuals that die) is the ratio of the transition rate from asymptomatic infection to KA to the total rate of progression to KA or recovery:

1 $$ \mathrm{Probability}\ \mathrm{of}\ \mathrm{developing}\ \mathrm{symptoms}=\frac{q_{23}}{q_{23}+{q}_{24}}. $$

### Model fitting

The model was fitted using the multi-state modelling package msm in R [44]. This package allows for the fact that some observations are exact (such as dates of death) and others are censored (such as the date of seroconversion) (see Additional file 1 for further details). The package estimates the transition intensity matrix *Q* and its confidence intervals by maximising the likelihood of the model given the data (see Additional file 1 for full details). The model also estimates the durations of the asymptomatic and symptomatic stages (the waiting times in states 2 and 3). The BFGS (Broyden-Fletcher-Goldfarb-Shanno) optimisation method (a quasi-Newtonian hill-climbing method that uses analytic derivatives for the optimisation [45]) in the optim function was used for finding the maximum likelihood. The confidence interval for the proportion of asymptomatic individuals that develop KA was calculated by bootstrap resampling of the data and refitting of the model with 1000 bootstrap samples [44].

## Results and discussion

### Delays to treatment

**Fig. 2:**
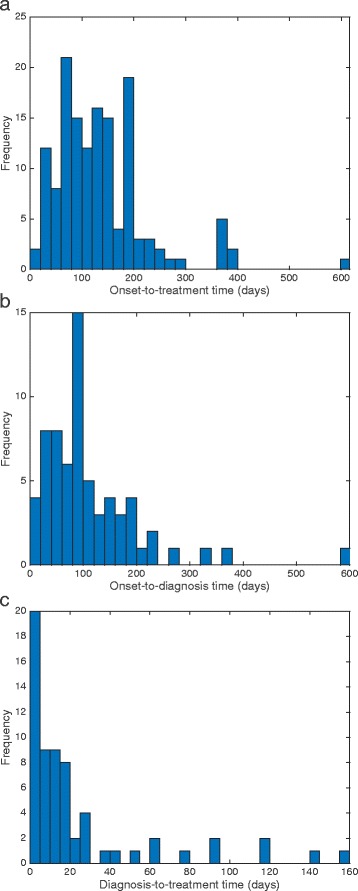
Delays to treatment. Distributions of (**a**) onset-to-treatment time, (**b**) onset-to-diagnosis time, and (**c**) diagnosis-to-treatment time. Sample sizes (n), medians, means and standard deviations (SDs): (**a**) n = 147, median = 120 days, mean = 133 days, SD = 90 days; (**b**) n = 67, median = 90 days, mean = 111 days, SD = 94 days; (**c**) n = 64, median = 12 days, mean = 24 days, SD = 35 days

Figure 2(a)-(c) show the distributions of onset-to-treatment, onset-to-diagnosis and diagnosis-to-treatment times for all KA patients for whom these times were recorded, along with the median, mean and standard deviation of each distribution. The median time of 120 days from onset to start of treatment is much longer than figures recently reported for Bangladesh (58 days) [20] and Nepal (55 days), but is comparable with those for Bihar (104 days) [41]. The discrepancy between our data and recently reported figures likely reflects the poorer state of the health care system and the greater cost of treatment in Bangladesh at the time, and the fact that there was no active detection programme for KA cases before 2005 [8, 46]. However, methodological differences between the studies may also account for some of the difference; for example, the dates of symptom onset and treatment from 1999 to 2002 were retrospectively ascertained in our study, so may be subject to recall bias.

### Risk of progression to KA

**Fig. 3:**
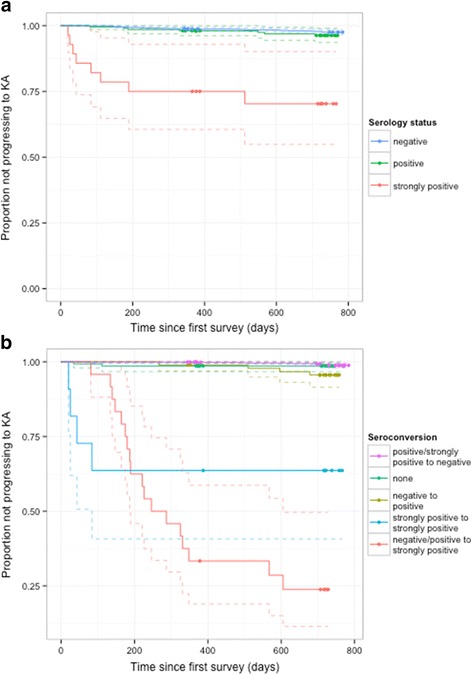
Kaplan-Meier curves for risk of progression to KA. Progression risk (with censoring) by (**a**) serology status at baseline, and (**b**) seroconversion from baseline survey (2002) to second survey (2003). Dots show where individuals were lost to follow-up; dashed lines show 95 % confidence intervals

In the analysis of the association between rK39 sero-status and progression to KA, 1,515 individuals who had not had KA previously and who had a serological measurement at baseline were included, amongst whom there were a total of 43 KA cases. The Kaplan-Meier curve in Fig. 3(a) illustrates that being highly seropositive at baseline was much more predictive of progression to clinical symptoms than moderate seropositivity or negative serology—29 % of strongly seropositive individuals progressed to KA compared to 3 % and 2 % of seropositive and seronegative individuals. Testing these associations using Cox regression modelling showed that there was relatively little difference in the risk of progression for seronegative and moderately seropositive individuals, but a much higher hazard ratio (HR) for those with high seropositivity (HR 17.7, 95 % CI 8.05-38.8, Table 3). This matches the analysis of Hasker et al. [28], which found hazard ratios for progression to KA ranging from 1.6 to 4.9 for seropositive individuals and from 7.7 to 39.6 for strongly seropositive individuals compared to seronegative individuals in 4 cohorts studied in Bihar, India and Terai, Nepal. However, the proportion of strongly seropositive individuals progressing to KA in the Bangladesh study was much higher than in all of the cohorts in [28] (29 % compared to 1.1-7.7 %) except for the TMRC cohort in Muzaffapur, Bihar that was selected based on high reported KA incidence in the year prior to the study (where the proportion was 23.3 %).

**Table 3:** Progression to KA depending on baseline rK39 sero-status. Hazard ratios and p-values estimated from fitted Cox proportional hazards regression models

Baseline sero-status	Total evaluated	Progressors (%)	Hazard ratio (95 % CI)	*p*
Seronegative, rK39 ELISA < 20CU	1,283	28 (2 %)	Ref.	N/A
Seropositive, 20CU ≤ rK39 ELISA < 61CU	204	7 (3 %)	1.61 (0.71–3.70)	0.26
Strongly seropositive, rK39 ELISA ≥ 61CU	28	8 (29 %)	17.7 (8.05–38.8)	8.4 × 10^−13^

As well as sero-status at baseline, seroconversion was an important marker for progression to KA. The seroconversion analysis was performed using 1,372 individuals that had rK39 ELISA readings from both the 2002 and 2003 surveys, 33 of whom developed KA. As expected from the figures shown in Table 3, a transition to strong seropositivity from either negative or moderately positive serology at baseline was associated with a high progression rate to KA compared with no seroconversion (HR 165, 95 % CI 74.6-365). Individuals that were strongly seropositive at both surveys also had a high risk of developing clinical symptoms (HR 61.5, 95 % CI 19.3-196). Seroconversion to moderate seropositivity was associated with an approximately 5-fold increase in risk of KA over no seroconversion, but the difference in progression for sero-deconvertors and non-convertors was not significant (Table 4). These results are similar to those for the highly endemic villages in Muzaffapur, Bihar in [28], which showed a hazard ratio for KA of 123.9 for individuals that became strongly seropositive compared with those that remained seronegative. However, a far greater proportion of high-titre seroconvertors progressed to KA in our data than in the previous study (13/18, 72 % as opposed to 9/37, 24.3 %). Also, unlike in this analysis, there was no significant association between moderate-titre seroconversion and progression to KA in the previous study. This is likely to be partly due to the differences, described in the Methods section, in the definitions of the cut-off values for seropositivity and strong seropositivity between the two studies.

**Table 4:** Progression to KA depending on change in serology status from first survey to second survey. Hazard ratios and p-values estimated from fitted Cox proportional hazard models. Sero-status: seronegative (−), seropositive (+), strongly seropositive (++)

Group	First survey	Second survey	Total evaluated	Progressors (%)	Hazard ratio (95 % CI)	*p*
Non-convertors	-	-	1103	10 (1 %)	Ref.	N/A
	+	+				
Sero-deconvertors	+/++	-	145	2 (1 %)	1.54 (0.34–7.02)	0.58
	++	+				
Seroconvertors	-	+	95	4 (4 %)	4.73 (1.48–15.1)	0.009
Strong seropositives	++	++	11	4 (36 %)	61.5 (19.3–196)	3.5 × 10^−12^
Strong seroconvertors	−/+	++	18	13 (72 %)	165 (74.6–365)	<2 × 10^−16^

The strong association between seroconversion to a high rK39 antibody titre and progression to KA in the study data suggests that rK39 ELISA could be used to predict KA prior to the onset of symptoms. However, since most high-titre seroconvertors who developed clinical symptoms did so before the second survey (Fig. 3(b)), more frequent testing would be required to use rK39 ELISA to pre-diagnose KA. Furthermore, even if practical constraints allowed for such testing, there is no suitable prophylactic treatment available at present, due to the toxicity of drugs currently used to treat KA.

### Natural history of VL

We first fitted the Markov model in its simplest form with constant transition intensities, i.e. treating individuals in the same state as having the same risk of infection and disease. The estimated transition intensity matrix for this 5-state model is

2 $$ Q=\left(\begin{array}{ccccc}\hfill -0.22\hfill & \hfill 0.21\hfill & \hfill 0\hfill & \hfill 0\hfill & \hfill 0.005\hfill \\ {}\hfill 0\hfill & \hfill -2.49\hfill & \hfill 0.36\hfill & \hfill 2.11\hfill & \hfill 0.02\hfill \\ {}\hfill 0\hfill & \hfill 0\hfill & \hfill -2.61\hfill & \hfill 2.48\hfill & \hfill 0.13\hfill \\ {}\hfill 0.31\hfill & \hfill 0\hfill & \hfill 0.01\hfill & \hfill -0.33\hfill & \hfill 0.006\hfill \\ {}\hfill 0\hfill & \hfill 0\hfill & \hfill 0\hfill & \hfill 0\hfill & \hfill 0\hfill \end{array}\right), $$

and the negative log-likelihood of the model is − log *L* = 1760.5 for the fitted intensities. The movement of individuals through the different disease states in the model with the estimated transition intensities is simulated in Additional file 2.

### Proportion of asymptomatic individuals that develop KA

The estimated intensities show that for asymptomatically infected individuals the probability of developing clinical symptoms (from (1)) was

$$ \frac{q_{23}}{q_{23}+{q}_{24}}=\frac{0.36}{0.36+2.11}=0.147, $$

with a 95 % bootstrap confidence interval (CI) of 0.126-0.200, and that recovery from KA with correct treatment was nearly 20 times as likely as dying from KA (*q*_34_/*q*_35_ = 19.5). This estimate for the proportion of asymptomatic individuals that develop KA, 14.7 % (95 % CI 12.6-20.0 %), is much larger than the figure of 0.33 % (95 % CI 0.22-0.49 %) estimated by Stauch et al. [33] from their SIRS model of VL transmission fitted to the KALANET trial data, and the figure of 4 % used in the transmission model of Medley et al. [20] based on the average progression to KA from baseline seropositivity in the KALANET data [28]. It is, however, similar to the proportion of DAT seroconvertors that developed KA in high-endemicity villages in the KALANET trial (10.1 %) [47], and the KA progression rate of asymptomatics identified by rK39 and PCR positivity in a study in two highly endemic villages in Bihar in 2005–2006 (23 %) [24]. It also agrees reasonably well with the 4:1 ratio of cases of seroconversion to KA reported by Bern et al. [31] for the data from 2002 to 2004. The variation in these estimates may reflect differences in various factors between the different study locations and periods, including parasite virulence, the immune and nutritional status of the host population, and the part of the epidemic curve the study population was on (incidence was climbing steeply in Fulbaria in 2000–2004 [7]). However, Stauch et al.’s estimate may be a considerable underestimate of the actual proportion that progress to KA due to the rapid cycling of individuals through asymptomatic infection in their model caused by fitting to cross-sectional data. It is likely, therefore, that Stauch et al. underestimate the contribution of KA patients to transmission relative to that of asymptomatic individuals.

### Average durations of asymptomatic infection and KA

The mean waiting times in the different disease states for the estimated transition intensities are shown in Table A2 in Additional file 1. The mean duration of asymptomatic infection was 147 days (95 % CI 130–166 days) and that of symptomatic infection was 140 days (95 % CI 123–160 days). Both these estimates are much longer than estimates from previous models. Stauch et al. [33] estimated that asymptomatic infection lasted on average 72 days (95 % CI 69–75 days) and that KA patients that received successful first-line treatment were cleared of parasites after 31 days and took 105 days on average from developing symptoms to become DAT negative and LST positive (i.e. to fully recover). Medley et al. [20] assumed that the duration of asymptomatic infection is 80 days in their model. Clearly, longer infection and disease durations can lead to increased transmission, as there are more opportunities for sandflies to become infected through feeding on humans. Based on our estimates, asymptomatic individuals are likely to contribute significantly to transmission even if their infectivity to sandflies is low relative to symptomatic individuals, due to the long asymptomatic infection period and the large ratio of asymptomatic to symptomatic individuals (although this proportion is smaller than that estimated by Stauch et al. [33]). If our estimates of the infection durations are representative of current high endemicity areas, more effort should be focused on reducing time to treatment through active case detection and early diagnosis, and greater surveillance of asymptomatic infection is required to identify individuals that are likely to develop KA and to estimate their contribution to transmission.

### Immunity

The estimated mean waiting time in the dormant/recovered stage was 1110 days (95 % CI 988–1247 days), which is much longer than that in the model of Stauch et al., where individuals remained DAT positive after asymptomatic infection or KA treatment for 74 days (95 % CI 65–84 days) and LST-positive for 307 days (95 % CI 260–356 days) on average, corresponding to a total time in the dormant/recovered stage of 381 days. We note that our estimate for the total time spent immune or with dormant infection is also likely to be an underestimate, due to the decrease in the sensitivity of the LST over the course of the study. This suggests that cellular immunity to the parasite can last for multiple years after asymptomatic infection or successful treatment for KA as reported elsewhere [48], rather than being lost within a year as suggested by Stauch et al. Indeed the age distribution of LST positivity in 2002 (when the LST had highest sensitivity) (Figure A1 in Additional file 1) shows an increase in the proportion of LST positive individuals with age, and Bern et al. [31] calculated from the data that there was a 48 % (95 % CI 38-59 %) increase in the chance of being LST positive with each 10-year increase in age, which suggests that cellular immunity wanes slowly. Of 530 individuals that tested LST positive in 2002, only one developed KA over the next two years, compared with 43 of the 1000 individuals that tested negative (relative risk = 0.04, 95 % CI 0.006-0.32), indicating that LST positivity represents effective immunity against KA. Given the increasing prevalence of LST positivity with age, its potentially long duration and the strong protection it offers against KA, control efforts should strive for 100 % detection and treatment of KA cases, particularly among individuals below 30 years of age who are less likely to be immune. The LST should also form a routine part of epidemiological studies to enable effective monitoring of levels of immunity within the population and better prediction of the risk of KA outbreaks, though this will require the production of sufficient quality leishmanin antigen to avoid issues associated with low antigen sensitivity [30].

### Model fit

To assess how well the multi-state model fits the data with the estimated transition intensity matrix in (2), we compared the observed number and prevalence of individuals in each state during the study period from 2002 to 2004 to the expected number and prevalence from the model. As shown in Table A7 and Figure A3 in Additional file 1, the overall fit of the model is good, with the observed and expected prevalences matching very closely for susceptible individuals, KA patients and recovered/dormant individuals, and fairly closely for asymptomatically infected individuals and dead individuals.

We also compared the model to a 6-state model in which asymptomatically infected individuals were split into two separate states—one for those who subsequently progressed to KA (‘pre-symptomatics’), and one for those who recovered without developing symptoms (‘asymptomatics’)—to determine whether there was a difference in the duration of asymptomatic infection for the two groups (see Additional file 1 for full details). Fitting the 6-state model to the data gave similar results to the 5-state model for the mean durations of the different disease stages (127 days for KA, 95 % CI 113–143 days, and 1108 days for the time spent immune or with dormant infection, 95 % CI 987–1244 days) and the proportion of infected individuals that develop symptoms (13.8 %, 95 % CI 9.7–19.4 %), and fairly similar asymptomatic infection durations of 135 days (95 % CI 109–167 days) and 159 days (95 % CI 138–183 days) for pre-symptomatics and asymptomatics.

### Covariates

A number of factors may be associated with altered KA risk, including sex, age and consistent use of bed nets (Table A3 and Figure A2 in Additional file 1). Individuals aged between 0 and 14 were at highest risk of KA (9.9 %), with a significantly decreased KA incidence in adults aged over 45, with only 2.8 % developing KA over the course of the study. Males were found to have a slightly higher incidence of KA than females (9.2 % compared to 7.8 %), while the use of bed nets more than halved the risk of KA (6.7 % compared to 14.7 %).

To further investigate the effects of these variables on risk of infection and disease, we fitted the model with each variable included as a covariate on the transition intensities. The results are summarised in Table A4 in Additional file 1, which gives hazard ratios for each covariate with 95 % confidence intervals. This analysis allows us to investigate which parts of the disease progression are affected by each covariate. For example, the hazard ratios for *q*_12_ and *q*_34_ for bed net use are 0.72 (95 % CI 0.52–1.00) and 1.44 (95 % CI 1.06–1.96), suggesting that bed net use reduces the risk of leishmanial infection by 28 % and increases the chance of recovery from KA by 44 % over no bed net use. This is potentially due to bed nets preventing infected sandflies from biting humans and either infecting or reinoculating them, and suggests that, with proper and widespread use, bed nets could form an effective part of VL control.

Performing a similar analysis on sex indicates that females have a lower rate of progression from asymptomatic infection to recovery/dormant infection (HR for *q*_24_ 0.73, 95 % CI 0.57–0.94) and a higher rate of return from recovery/dormant infection to susceptibility (HR for *q*_41_ 1.36, 95 % CI 1.07–1.72). While Table A3 and Figure A2 in Additional file 1 suggest that the risk of KA generally decreases with age, the risk does not decrease linearly. Individuals aged 15–45 appear to be at increased risk of infection compared with those aged 0–14 (HR for *q*_12_ 1.31, 95 % CI 0.99–1.73), but more likely to recover from asymptomatic infection without developing KA (HR for *q*_24_ 1.35, 95 % CI 1.03-1.77) and less likely to recover from KA (HR *q*_34_ 0.75, 95 % CI 0.56-1.00). The risk of death from KA is higher for adults aged over 45 than children aged 0–14 (HR for *q*_35_ 5.19, 95 % CI 1.28–21.0). As expected, the risk of death due to other causes is much higher for adults aged over 45 than children aged 0–14 (HRs for *q*_15_, *q*_25_ and *q*_45_ 15.9, 95 % CI 4.5-55.4). Table A5 in Additional file 1 shows the probability of developing KA from asymptomatic infection for the different groups for each covariate. The probability is fairly similar across all groups and covariates, at approximately 0.15-0.16, apart from for 0–14-year-olds, who have a higher probability of developing symptoms of 0.17, and those aged over 45, who have a much lower probability of symptoms of 0.06. These differences have implications for design of surveillance systems; for example, they suggest that children are likely to be a more sensitive indicator of continued transmission, whereas most infection in adults is asymptomatic. Comparison of the model with each of the covariates to the model without any covariates using the likelihood ratio test and Akaike information criterion (Table A6 in Additional file 1) reveals that including each of the covariates significantly improves the fit of the model to the data. The largest improvement in the fit is given by including age-group as a covariate, which decreases the negative log-likelihood for the model to − log *L* = 1720.7 (*p* = 1.8 × 10^−10^ for the likelihood ratio test against the model with no covariates).

## Conclusions

By reanalysing a detailed dataset on the development of clinical VL in Bangladesh in 2002–2004, we have been able to provide an independent estimate for the proportion of asymptomatically infected individuals who progress to KA of 14.7 % and an estimate for the asymptomatic infection period of 147 days. Both these estimates are similar to those reported in the literature by other means [32, 34], but much higher and longer respectively than those used in the main previous modelling studies [20, 33, 49].

Our analysis also shows that high rK39 levels, and in particular seroconversion to a high rK39 titre, are good predictors of progression to clinical VL, providing independent support for the results from a previous study [28]. This suggests that it may be possible to screen individuals to identify those who are likely to progress to clinical VL, improve their access to treatment and potentially reduce their infectious period and onward transmission through targeted IRS.

The role of seroconverting and symptomatic individuals in transmission depends not only on the proportion of individuals in each state and the lengths of time they are in each state, but also on their infectivity to sand flies. The relative infectivity of asymptomatics and symptomatics has rarely been studied, [50, 51], although one xenodiagnostic study in Ethiopian patients and vectors suggests that there may be marked changes in infectivity with parasitaemia [52], a measure which was not noted in this study.

We have also highlighted that the time from onset of symptoms to treatment in this area of Bangladesh in the early 2000s was considerably longer than in recent times [41]. During the 2002–2004 study period, Bangladesh experienced a major shortage of sodium stibogluconate, the only KA treatment drug in use at the time [53]. The shortage led to lack of supply in government health facilities and price gouging in the private marketplace [54]. At the time the study began, the median time from onset to treatment was 6 months and the only available drug was provided by the project; this shortened to 3–4 months over the course of the study. Improvements in drug availability after 2005, and especially after the implementation of active case detection and treatment with short course liposomal amphotericin B in Fulbaria [55], may have helped to drive reductions in incidence in Bangladesh by shortening the period symptomatic individuals spent in the community prior to treatment [20].

Despite our limited understanding of the natural history of VL [26], there are only two detailed epidemiological studies of the progression of leishmanial infection and disease. If we are to further refine control strategies to bring VL to local elimination, such studies will be invaluable, particularly if they can assist in identifying individuals who will develop KA or who may contribute most substantially to transmission.

Our intention is to use the results of our statistical modelling to develop transmission dynamics models of VL to evaluate the effect of potential interventions and the feasibility of achieving the 2020 elimination goals. At present, our estimate of the rate of infection, *q*_12_, is independent of time and of the prevalence of infection, but should be a function of the number of infectious sandflies, which itself is dependent on the infectiousness of the human population. Within such a framework, we can also consider the spatial kernel of transmission and the impact of individual circumstances such as livestock ownership, nutritional status and sleeping location. To refine our estimates of the asymptomatic infection period and proportion of infected individuals that develop KA, and to assess the sensitivity and specificity of the rK39 ELISA and LST used in the study, we need to account for misclassification of individuals’ disease states in the multi-state model due to errors in the test results. This can be achieved using a hidden Markov model, in which individuals’ observed states can be misclassifications of their true, underlying disease states.

The results of this study and the potential for future development highlight the importance of detailed, longitudinal studies for improving understanding of VL and creating datasets that can be used for the design of interventions. As our understanding of the disease develops, the requirements for such data change, indicating that datasets such as these must be continually gathered.

## Additional files

Additional file 1:
**Further explanation of the multi-state Markov model and fitting, tables of state classifications and results from the model, and comparison of the model with a 6-state model that splits the asymptomatic group into those that progress to KA and those that do not.** (PDF 301 kb) Additional file 2:
**Matlab file for numerically solving the system of ordinary differential equations (ODEs) that can be used to represent the 5-state model for a given set of parameter values.** (M 1 kb)

## References

[1] Boelaert M, Bhattacharya S, Chappuis F, El Safi SH, Hailu A, Mondal D (2007). Evaluation of rapid diagnostic tests: Visceral leishmaniasis. Nat Rev Microbiol.

[2] Chappuis F, Sundar S, Hailu A, Ghalib H, Rijal S, Peeling RW (2007). Visceral leishmaniasis: What are the needs for diagnosis, treatment and control?. Nat Rev Microbiol.

[3] Maia Z, Lírio M, Mistro S, Mendes C, Mehta SR, Badaro R (2012). Comparative study of rK39 leishmania antigen for serodiagnosis of visceral leishmaniasis: Systematic review with meta-analysis. PLoS Negl Trop Dis.

[4] de Ruiter C, Van der Veer C, Leeflang M, Deborggraeve S, Lucas C, Adams E (2014). Molecular tools for diagnosis of visceral leishmaniasis: Systematic review and meta-analysis of diagnostic test accuracy. J Clin Microbiol.

[5] Boelaert M, Verdonck K, Menten J, Sunyoto T, van Griensven J, Chappuis F (2014). Rapid tests for the diagnosis of visceral leishmaniasis in patients with suspected disease.

[6] Dye C, Wolpert DM (1988). Earthquakes, influenza and cycles of Indian kala-azar. Trans R Soc Trop Med Hyg.

[7] Islam S, Kenah E, Bhuiyan MAA, Rahman KM, Goodhew B, Ghalib CM (2013). Clinical and immunological aspects of post-kala-azar dermal leishmaniasis in Bangladesh. Am J Trop Med Hyg.

[8] Chowdhury R, Mondal D, Chowdhury V, Faria S, Alvar J, Nabi SG (2014). How far are we from visceral leishmaniasis elimination in Bangladesh? An assessment of epidemiological surveillance data. PLoS Negl Trop Dis.

[9] Rijal S (2010). WHO Visceral Leishmaniasis country data.

[10] Bhunia GS, Kesari S, Chatterjee N, Kumar V, Das P. The burden of visceral leishmaniasis in India: Challenges in using remote sensing and GIS to understand and control. ISRN Infect Dis. 2012. doi:10.5402/2013/675846.

[11] Mondal D, Singh SP, Kumar N, Joshi A, Sundar S, Das P (2009). Visceral leishmaniasis elimination programme in India, Bangladesh, and Nepal: Reshaping the case finding/Case management strategy. PLoS Negl Trop Dis.

[12] Sharma SN, Batthacharya S, Sundar S (2010). WHO Visceral Leishmaniasis country data.

[13] Regional strategic framework for elimination of kala-azar from the South-East Asia region (2005–2015). New Delhi: WHO Regional Office for South-East Asia. World Health Organization; 2005.

[14] Sundar S, Chakravarty J, Fong I (2013). Leishmaniasis: Challenges in the control and eradication. Challenges in infectious diseases.

[15] Chowdhury R, Huda M, Kumar V, Das P, Joshi A, Banjara M (2011). The Indian and Nepalese programmes of indoor residual spraying for the elimination of visceral leishmaniasis: Performance and effectiveness. Ann Trop Med Parasitol.

[16] Coleman M, Foster GM, Deb R, Singh RP, Ismail HM, Shivam P (2015). DDT-based indoor residual spraying suboptimal for visceral leishmaniasis elimination in India. Proc Natl Acad Sci.

[17] Hasker E, Singh SP, Malaviya P, Picado A, Gidwani K, Singh RP (2012). Visceral leishmaniasis in rural Bihar. India Emerg Infect Dis.

[18] Chowdhury R, Dotson E, Blackstock AJ, McClintock S, Maheswary NP, Faria S (2011). Comparison of insecticide-treated nets and indoor residual spraying to control the vector of visceral leishmaniasis in Mymensingh district, Bangladesh. Am J Trop Med Hyg.

[19] Rutte EA le, Coffeng LE, Bontje DM, Hasker Epco C, Postigo JAR, Dagne DA, et al. Feasibility of eliminating visceral leishmaniasis from the Indian subcontinent: Explorations with a deterministic transmission model. Parasites and Vectors (Submitted). 2015. 10.1186/s13071-016-1292-0 PMC4717541 26787302

[20] Medley GF, Hollingsworth TD, Olliaro PL, Adams ER. Visceral leishmaniasis control: Health-seeking, diagnostics and transmission. Nature supplement (Under review). 2015. 10.1038/nature16042 26633763

[21] Malaviya P, Picado A, Singh S, Hasker E, Singh R, Boelaert M (2010). Visceral leishmaniasis in Muzaffarpur district, Bihar, India from 1990 to 2008. PLoS One.

[22] Alvar J, Vélez ID, Bern C, Herrero M, Desjeux P, Cano J (2012). Leishmaniasis worldwide and global estimates of its incidence. PLoS One.

[23] Singh VP, Ranjan A, Topno RK, Verma RB, Siddique NA, Ravidas VN (2010). Estimation of under-reporting of visceral leishmaniasis cases in Bihar. India Am J Trop Med Hyg.

[24] Das V, Siddiqui N, Verma R, Topno R, Singh D, Das S (2011). Asymptomatic infection of visceral leishmaniasis in hyperendemic areas of Vaishali district, Bihar, India: A challenge to kala-azar elimination programmes. Trans R Soc Trop Med Hyg.

[25] Das S, Matlashewski G, Bhunia GS, Kesari S, Das P (2014). Asymptomatic Leishmania infections in northern India: A threat for the elimination programme?. Trans R Soc Trop Med Hyg.

[26] Rock KS, Rutte EA le, Vlas SJ de, Adams ER, Medley GF, Hollingsworth TD. Uniting mathematics and biology for control of visceral leishmaniasis. Trends Parasitol. 2015 10.1016/j.pt.2015.03.007 25913079

[27] Picado A, Kumar V, Das M, Burniston I, Roy L, Suman R (2009). Effect of untreated bed nets on blood-fed Phlebotomus argentipes in kala-azar endemic foci in Nepal and India. Mem Inst Oswaldo Cruz.

[28] Hasker E, Malaviya P, Gidwani K, Picado A, Ostyn B, Kansal S, et al. Strong association between serological status and probability of progression to clinical visceral leishmaniasis in prospective cohort studies in India and Nepal. PLoS Negl Trop Dis. 2014;8(1). 10.1371/journal.pntd.0002657 PMC3900391 24466361

[29] Bern C, Hightower A, Chowdhury R, Ali M, Amann J, Wagatsuma Y (2005). Risk factors for kala-azar in Bangladesh. Emerg Infect Dis.

[30] Bern C, Amann J, Haque R, Chowdhury R, Ali M, Kurkjian KM (2006). Loss of leishmanin skin test antigen sensitivity and potency in a longitudinal study of visceral leishmaniasis in Bangladesh. Am J Trop Med Hyg.

[31] Bern C, Haque R, Chowdhury R, Ali M, Kurkjian KM, Vaz L (2007). The epidemiology of visceral leishmaniasis and asymptomatic leishmanial infection in a highly endemic Bangladeshi village. Am J Trop Med Hyg.

[32] Rees PH, Kager PA. Visceral leishmaniasis and post-kala-azar dermal leishmaniasis. In: Peters W, Killick-Kendrick R, editors. The leishmaniases in biology and medicine. Volume II. Clinical aspects and control. London: Academic Press; 1987. p. 583–615.

[33] Stauch A, Sarkar RR, Picado A, Ostyn B, Sundar S, Rijal S (2011). Visceral leishmaniasis in the Indian subcontinent: Modelling epidemiology and control. PLoS Negl Trop Dis.

[34] Mubayi A, Castillo-Chavez C, Chowell G, Kribs-Zaleta C, Siddiqui NA, Kumar N (2010). Transmission dynamics and underreporting of kala-azar in the Indian state of Bihar. J Theor Biol.

[35] Houghton RL, Petrescu M, Benson DR, Skeiky YA, Scalone A, Badaró R (1998). A cloned antigen (recombinant k39) of Leishmania chagasi diagnostic for visceral leishmaniasis in human immunodeficiency virus type 1 patients and a prognostic indicator for monitoring patients undergoing drug therapy. J Infect Dis.

[36] Badaro R, Benson D, Eulalio M, Freire M, Cunha S, Netto E (1996). RK39: A cloned antigen of Leishmania chagasi that predicts active visceral leishmaniasis. J Infect Dis.

[37] Kurkjian K, Vaz L, Haque R, Cetre-Sossah C, Akhter S, Roy S (2005). Application of an improved method for the recombinant K39 enzyme-linked immunosorbent assay to detect visceral leishmaniasis disease and infection in Bangladesh. Clin Diagn Lab Immunol Am Soc Microbiol.

[38] Sokal JE (1975). Editorial: Measurement of delayed skin-test responses. N Engl J Med.

[39] Gramiccia M, Bettini S, Gradoni L, Ciarmoli P, Verrilli M, Loddo S (1990). Leishmaniasis in Sardinia 5. Leishmanin reaction in the human population of a focus of low endemicity of canine leishmaniasis. Trans R Soc Trop Med Hyg.

[40] Weigle KA, Valderrama L, Arias AL, Santrich C, Saravia NG (1991). Leishmanin skin test standardization and evaluation of safety, dose, storage, longevity of reaction and sensitization. Am J Trop Med Hyg.

[41] Boettcher JP, Siwakoti Y, Milojkovic A, Siddiqui NA, Gurung CK, Rijal S (2015). Visceral leishmaniasis diagnosis and reporting delays as an obstacle to timely response actions in Nepal and India. BMC Infect Dis.

[42] Picado A, Singh SP, Rijal S, Sundar S, Ostyn B, Chappuis F (2010). Longlasting insecticidal nets for prevention of Leishmania donovani infection in India and Nepal: Paired cluster randomised trial. BMJ.

[43] Hasker E, Kansal S, Malaviya P, Gidwani K, Picado A, Singh RP (2013). Latent infection with Leishmania donovani in highly endemic villages in Bihar, India. PLoS Negl Trop Dis.

[44] Jackson C. Multi-state modelling with R: the msm package. MRC Biostatistics Unit. 2014. https://cran.r-project.org/web/packages/msm/vignettes/msm-manual.pdf. Accessed 1 Jul 2015.

[45] Fletcher R. Practical methods of optimization. Chichester: John Wiley & Sons; 1987

[46] Das A, Harries A, Hinderaker S, Zachariah R, Ahmed B, Shah G (2014). Active and passive case detection strategies for the control of leishmaniasis in Bangladesh. Public Health Action.

[47] Ostyn B, Gidwani K, Khanal B, Picado A, Chappuis F, Singh S (2011). Incidence of symptomatic and asymptomatic Leishmania donovani infections in high-endemic foci in India and Nepal: A prospective study. PLoS Negl Trop Dis.

[48] Pampiglione S, Manson-Bahr P, La Placa M, Borgatti M, Musumeci S (1975). Studies in mediterranean leishmaniasis: 3. The leishmanin skin test in kala-azar. Trans R Soc Trop Med Hyg.

[49] Stauch A, Duerr H-P, Picado A, Ostyn B, Sundar S, Rijal S (2014). Model-based investigations of different vector-related intervention strategies to eliminate visceral leishmaniasis on the Indian subcontinent. PLoS Negl Trop Dis.

[50] Knowles R, Napier LE, Smith R (1926). On a herpetomonas found in the gut of the sandfly Phlebotomus argentipes, fed on kala-azar patients: A preliminary note.

[51] Shortt HE, Craighead AC, Barraud PJ, K-a Commission K-a et al. Note on a massive infection of the pharynx of Phlebotomus argentipes with herpetomonas donovani. Indian J Med Res. 1926;13(3):441-4.

[52] Miller E, Warburg A, Novikov I, Hailu A, Volf P, Seblova V (2014). Quantifying the contribution of hosts with different parasite concentrations to the transmission of visceral leishmaniasis in Ethiopia. PLoS Negl Trop Dis.

[53] Ahluwalia IB, Bern C, Costa C, Akter T, Chowdhury R, Ali M (2003). Visceral leishmaniasis: Consequences of a neglected disease in a Bangladeshi community. Am J Trop Med Hyg.

[54] Ozaki M, Islam S, Rahman KM, Rahman A, Luby SP, Bern C (2011). Economic consequences of post–kala-azar dermal leishmaniasis in a rural Bangladeshi community. Am J Trop Med Hyg.

[55] Lucero E, Collin SM, Gomes S, Akter F, Asad A, Kumar Das A (2015). Effectiveness and safety of short course liposomal amphotericin B (amBisome) as first line treatment for visceral leishmaniasis in Bangladesh. PLoS Negl Trop Dis.

