# Supplementary material for: Quantification of the natural history of visceral leishmaniasis and consequences for control
Source: Parasit Vectors. 2015 Oct 22;8:521. doi: 10.1186/s13071-015-1136-3 (PMC4618734; doi:10.1186/s13071-015-1136-3)
Supplement: Additional file 1: — Further explanation of the multi-state Markov model and fitting, tables of state classifications and results from the model, and comparison of the model with a 6-state model that splits the asymptomatic group into those that progress to KA and those that do not. (PDF 301 kb) [file 13071_2015_1136_MOESM1_ESM.pdf]

Chapman *et al. Parasites & Vectors*
DOI 10.1186/s13071-015-1136-3

RESEARCH

Quantification of the natural history of visceral leishmaniasis and consequences for control: Additional file 1

Lloyd A.C. Chapman[1*], Louise Dyson[1], Orin Courtenay[1], Rajib Chowdhury[2,3], Caryn Bern[4], Graham F. Medley[5†] and T. Deirdre Hollingsworth[1†]

[*]Correspondence:
L.Chapman.1@warwick.ac.uk
[1]School of Life Sciences, University of Warwick, Gibbet Hill Campus, Coventry CV4 7AL , UK
Full list of author information is available at the end of the article
[†]Equal contributor

Multi-state Markov model of natural history of visceral leishmaniasis

A general continuous-time multi-state Markov model of disease progression consists of R disease states and M individuals, each of whom is in one of the R states at any particular time. The state occupied by the ith individual at time t is denoted by $S_i(t)$ and the movement of individuals between the states is governed by a set of transition intensities $q_{rs}(t,z)$ $(r,s=1,\dots,R)$, which may depend on time and a set of (potentially individual-specific) explanatory variables z. The transition intensity q_{rs} represents the instantaneous risk of moving from state r to state s for $r \neq s$,

$$q_{rs}(t,z) = \lim_{\delta t \to 0} Pr(S_i(t+\delta t) = s | S_i(t) = r)/\delta t,$$

and $q_{rr} := -\sum_{s \neq r} q_{rs}$. The intensities form an $R \times R$ matrix, Q, whose rows sum to zero. Fitting the multi-state model to observations of individuals' disease states enables Q to be estimated.

The Markov assumption is that the future state of the system depends only on its current state and not its history, i.e. $q_{rs}(t,z,\mathcal{F}_t) = q_{rs}(t,z)$, where $\mathcal{F}_t$ is the history of the process before time t. This is equivalent to assuming that all individuals in a state have the same expected outcome regardless of their previous states; for example, an individual with asymptomatic infection will progress to KA with the same probability regardless of whether this is their first or second infection.

Likelihood for multi-state model

The likelihood for the multi-state model given the data is calculated from the transition probability matrix $P(u,t)$, whose $(r,s)^{\text{th}}$ entry $p_{rs}(u,t)$ is the probability of being in state s at time $t > u$, given that the state at time u was r (note that the process may have passed through other states between times u and t). $P(u,t)$ is calculated from the forward Kolmogorov equations [1]:

$$\frac{\partial P(u,t)}{\partial t} = P(u,t)Q(t) \tag{A1}$$

For time-homogeneous Markov processes such as the models we consider (in which the transition intensities q_{rs} are independent of t), $P(u,t) = P(t-u)$, and $P(t-u)$

may be calculated by the matrix exponential

$$P = \exp((t-u)Q), \tag{A2}$$

which is the solution, in matrix form, of (A1). If the data for the ith individual consists of a series of n_i observation times $(t_{i1}, t_{i2}, \ldots, t_{in_i})$ and corresponding disease states $(S_i(t_{i1}), S_i(t_{i2}), \ldots, S_i(t_{in_i}))$, the contribution to the likelihood from each pair of successive observed states is

$$L_{i,j} = p_{S_i(t_{ij})S_i(t_{i,j+1})}(t_{i,j+1} - t_{ij}), \quad i = 1, \ldots, M,\ j = 1, \ldots, n_i, \tag{A3}$$

which is the $(S_i(t_{ij}), S_i(t_{i,j+1}))^{\text{th}}$ entry of the transition probability matrix $P(t)$ evaluated at $t = t_{i,j+1} - t_{ij}$. The full likelihood $L(Q)$ is given by the product of all such $L_{i,j}$ over all M individuals and all transitions

$$L(Q) = \prod_{i=1}^{M} \prod_{j=1}^{n_i} L_{i,j} = \prod_{i=1}^{M} \prod_{j=1}^{n_i} p_{S_i(t_{ij})S_i(t_{i,j+1})}(t_{i,j+1} - t_{ij}). \tag{A4}$$

The transition intensity matrix Q is estimated by maximising the likelihood in (A4) as a function of Q.

Exactly observed transition times

Equation (A4) gives the likelihood for the model when all observations of individuals' disease states are at arbitrary times. However, some observations in the dataset can be regarded as being at exact transition times with no transitions having occurred since the last observation, such as the date of the end of KA treatment, which is preceded by the observation of the onset of symptoms. For such observations the contribution to the likelihood is

$$L_{i,j} = \exp(q_{S_i(t_{ij})S_i(t_{i,j+1})}(t_{i,j+1} - t_{ij}))q_{S_i(t_{ij})S_i(t_{i,j+1})},$$

since the individual is in state $S_i(t_{ij})$ throughout $[t_{ij}, t_{i,j+1}]$ and then enters state $S_i(t_{i,j+1})$ at $t_{i,j+1}$.

Censored observations

The contribution to the likelihood from each pair of successive states given in (A3) applies when individual i is *known* to be in state $S_i(t_{i,j+1})$ at time $t_{i,j+1}$. However, for some observations in the dataset the individual is known only to be in a certain set of states, since either the ELISA or LST test is missing. For such an observation $S_i(t_{i,j+1})$, known to be in the set of states C, the contribution to the likelihood is

$$L_{i,j} = \sum_{m \in C} p_{S_i(t_{ij})m}(t_{i,j+1} - t_{ij}).$$

Covariates

The dependence of infection and disease progression on characteristics of individuals such as sex and age is potentially very important in designing control interventions for VL. Individual-specific covariates can be incorporated into the multi-state model in the form of a proportional hazards model, by replacing the transition intensity matrix elements by

$$q_{rs}(z_i) = q_{rs}^{(0)} \exp\left(\beta_{rs} z_i\right), \tag{A5}$$

where $q_{rs}^{(0)}$ are the baseline transition intensities, z_i is the value of the covariate z for the ith individual, and β_{rs} represents the effect of the covariate ($\exp(\beta_{rs})$ is the hazard ratio for a unit increase in z). The modified transition intensities are then used to determine the likelihood, and the likelihood is maximised over the baseline intensities $q_{rs}(0)$ and log-hazard ratios β_{rs}.

Classification of states

The full classification of observations in the data into the different states in the 5-state Markov model, including censored states for missing rK39 ELISA and LST readings is given in Table A1.

Table A1: Full classification of individuals into different disease states in 5-state model of natural history.

Disease/censored state	Description	rK39 ELISA	LST	KA	Post KA	Previous rK39 ELISA
1	Susceptible	−	−	−		−
2	Asymptomatic infected	+	−	−	−	+/−
3	Symptomatic infected (KA)	+	−	+		+/−
4	Recovered/Dormant	−	−/?	−		+
		+	−/?	−	+	
		?	−	−	+	+
		+/−/?	+	−		
5	Dead	NA	NA	NA	NA	NA
91 (1 or 4)	Susceptible/recovered	−	?	−		−/?
		?	−	−	+	−/?
92 (2 or 4)	Asymptomatic/ recovered	+	?	−	−	
		?	−	−	−	+
93 (1 or 2)	Susceptible/asymptomatic	?	−	−	−	−

Key: + positive for this marker, − negative for this marker, ? missing test

Results tables for 5-state Markov model

Table A2: Mean durations of different disease stages in 5-state Markov model determined by maximum likelihood estimation.

Disease state	Mean waiting time (95% CI) (days)
Susceptible	1696 (1492–1927)
Asymptomatic	147 (130–166)
KA	140 (123–160)
Recovered/Dormant	1110 (988–1247)

DOI 10.1186/s13071-015-1136-3

Table A3: Cumulative incidence of kala-azar (KA) by sex from 1999-2004 for different age groups and bed net use. Individuals with no serology data were excluded; n = number of individuals in each group.

		Male		Female		Both	
		n	KA (%)	n	KA (%)	n	KA (%)
Age (years)	0-14	434	47 (10.8%)	469	42 (9.0%)	903	89 (9.9%)
	15-45	448	40 (8.9%)	553	46 (8.3%)	1001	86 (8.6%)
	> 45	123	6 (4.9%)	124	1 (0.8%)	247	7 (2.8%)
All		1006	93 (9.2%)	1146	89 (7.8%)	2152	182 (8.5%)
Bed net use	Little/no use	154	23 (14.9%)	199	29 (14.6%)	353	52 (14.7%)
	Consistent use	805	62 (7.7%)	871	50 (5.7%)	1676	112 (6.7%)

Table A4: Hazard ratios (HR) and 95% confidence intervals (CI) for transition intensities dependent on age-group, sex and bed net use. HRs and CIs estimated by fitting proportional hazard models for the intensities (equation (A5)) by maximum likelihood estimation.

Transition intensity	Age (years) (Ref.: 0-14)				Sex (Ref.: Male)		Bed net use (Ref: No use)	
	15-45		>45					
	HR	95% CI	HR	95% CI	HR	95% CI	HR	95% CI
q_{12}	1.31	(0.99–1.73)	1.41	(0.92–2.16)	1.00	(0.77–1.31)	0.72	(0.52–1.00)
q_{15}	$0.28^{\dagger}$	(0.02–3.10)	$15.9^{\dagger}$	(4.53–55.4)	0.66	(0.14–3.11)	$1^{\ddagger}$	N/A
q_{23}	1.16	(0.69–1.95)	0.40	(0.06–2.64)	0.75	(0.45–1.25)	0.99	(0.58–1.68)
q_{24}	1.35	(1.03–1.77)	1.39	(0.88–2.19)	0.73	(0.57–0.94)	1.02	(0.76–1.36)
q_{25}	$0.28^{\dagger}$	(0.02–3.10)	$15.9^{\dagger}$	(4.53–55.4)	0.67	(0.10–4.32)	$1^{\ddagger}$	N/A
q_{34}	0.75	(0.56–1.00)	1.59	(0.78–3.21)	0.83	(0.63–1.10)	1.44	(1.06–1.96)
q_{35}	0.69	(0.16–2.88)	5.19	(1.28–21.0)	1.55	(0.46–5.29)	$1^{\ddagger}$	N/A
q_{41}	0.83	(0.63–1.08)	1.11	(0.80–1.54)	1.36	(1.07–1.72)	0.94	(0.71–1.26)
q_{43}	0.31	(0.07–1.38)	0.73	(0.12–4.53)	0.58	(0.14–2.40)	0.80	(0.09–6.73)
q_{45}	$0.28^{\dagger}$	(0.02–3.10)	$15.9^{\dagger}$	(4.53–55.4)	1.42	(0.29–6.96)	$1^{\ddagger}$	N/A

† Age effect constrained to be equal for death rates for each age group.

‡ Death rates assumed to be independent of bed net use.

Table A5: Probability of progressing to KA from asymptomatic infection for different groups for each covariate.

Covariate	Group	Probability of developing symptoms
None	N/A	0.147
Age (years)	0-14	0.170
	15-45	0.151
	>45	0.056
Sex	Male	0.147
	Female	0.150
Bed net use	Little/no use	0.159
	Consistent use	0.155

Table A6: Comparison of model without covariates to model with each covariate included, by the likelihood ratio test and Akaike Information Criterion (AIC).

Covariate	Negative log-likelihood, $-\log L$	Likelihood ratio statistic, $D = -2\log(L_0/L_1)$	Difference in degrees of freedom, $df_1 - df_0$	p	AIC
None	1760.5	N/A	N/A	N/A	3541.1
Age	1720.7	79.8	16	1.8×10^{-10}	3493.3
Sex	1749.2	22.7	10	0.01	3538.4
Bed net use	1745.5	30.0	6	3.9×10^{-5}	3523.1

AIC $= 2k - 2\log L$, where k is the number of fitted parameters in the model.

0 denotes model with no covariates, 1 denotes model with covariate.

DOI 10.1186/s13071-015-1136-3

Figure A1: Proportion of individuals with a positive leishmanin skin test (LST) by age group in 2002.

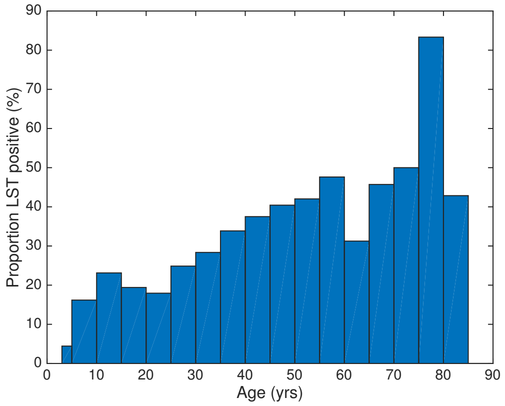

Figure A2: Cumulative incidence of KA from 1999-2004 by age group.

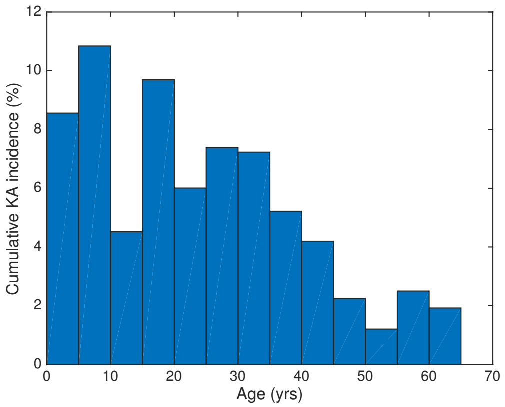

Model fit

To assess the goodness of fit of the multi-state model without covariates, we calculated the observed and expected numbers and percentages of individuals (prevalences) in each state for the study period from 2002-2004 at 3-month intervals (see Table A7). The expected number of individuals in each state at time t was calculated by multiplying the number of individuals under observation at time t by the initial proportion of individuals in state r and the transition probability matrix for the time interval t, $P(t)$. Table A7 and Figure A3 show that the observed and expected numbers and prevalences match closely for susceptible individuals, KA patients and recovered/dormant individuals (states 1, 3 and 4), but that the predicted number of asymptomatically infected individuals (state 2) is underestimated by the model and the number of deaths (state 5) is over-estimated. The discrepancy in the number of asymptomatic individuals is likely due to variation in the transition rates with time (e.g. the infection rate q_{12} changing as asymptomatic infection and KA incidence changed during the study period), which is not accounted for in

DOI 10.1186/s13071-015-1136-3

the model; while the discrepancy in the number of deaths is likely due to the large number of individuals that died who are not included in the model fitting (37 out of 64) as their only observation is their date of death.

Table A7: Observed numbers of individuals in each state during the 2002-2004 study period and expected numbers from the model.

Time since start of 2002 (years)		State 1	2	3	4	5	Total
0.2	Observed	858	149	5	591	1	1604
	Expected	877.2	74.0	12.9	604.0	35.8	1604
0.45	Observed	856	141	7	597	4	1605
	Expected	877.6265	74.1	12.9	601.8	38.6	1605
0.7	Observed	847	138	17	598	6	1606
	Expected	877.8	74.1	12.9	599.8	41.4	1606
0.95	Observed	841	137	15	600	12	1605
	Expected	876.7	74.1	12.9	597.3	44.1	1605
1.2	Observed	929	129	11	630	15	1714
	Expected	935.6	79.1	13.7	635.7	50.0	1714
1.45	Observed	936	123	9	632	19	1719
	Expected	937.5	79.2	13.7	635.5	53.0	1719
1.7	Observed	933	119	11	635	22	1720
	Expected	937.1	79.2	13.7	634.0	56.0	1720
1.95	Observed	930	117	9	639	24	1719
	Expected	935.5	79.1	13.7	631.8	58.8	1719

Figure A3: Observed and expected prevalence of each state during the 2002-2004 study period.

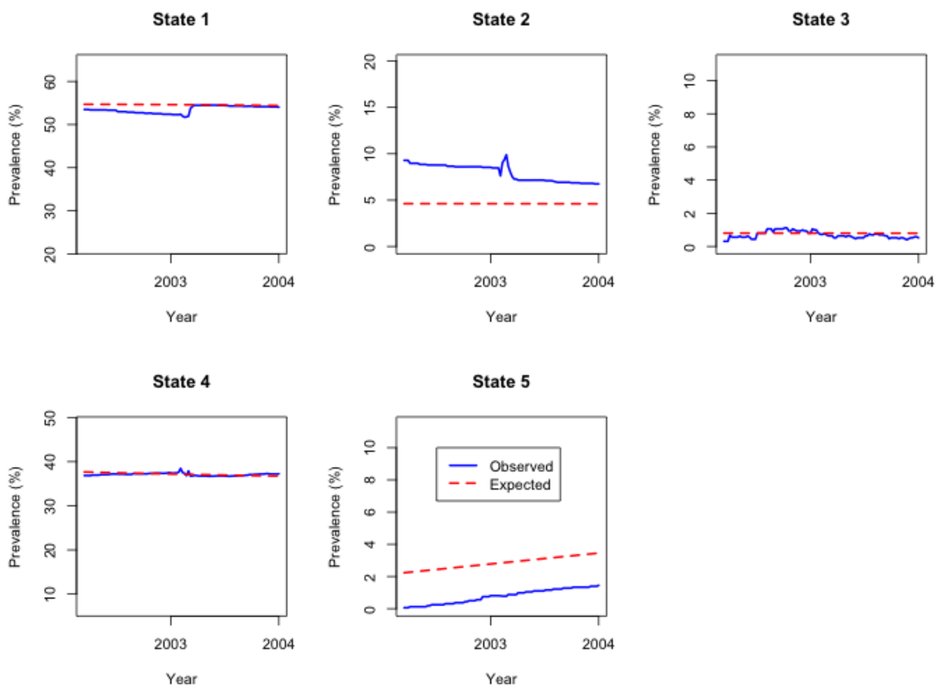

DOI 10.1186/s13071-015-1136-3

Comparison with model with separate states for asymptomatic and pre-symptomatic infection

To assess whether there is a difference in the duration of asymptomatic infection for individuals who progress to KA (referred to as pre-symptomatics) and those who recover without developing symptoms (referred to as asymptomatics from here on), and whether this affects the estimate of the proportion of infected individuals that develop KA, we fitted the 6-state model shown in Figure A4 with separate states for pre-symptomatics (state 2) and asymptomatics (state 6) to the data. The state space of the model was otherwise the same as in the 5-state model. The transition intensity matrix for this model is

$$Q_A = \begin{pmatrix} q_{11} & q_{12} & 0 & 0 & q_{15} & q_{16} \\ 0 & q_{22} & q_{23} & 0 & 0 & 0 \\ 0 & 0 & q_{33} & q_{34} & q_{35} & 0 \\ q_{41} & 0 & q_{43} & q_{44} & q_{45} & 0 \\ 0 & 0 & 0 & 0 & 0 & 0 \\ 0 & 0 & 0 & q_{64} & q_{65} & q_{66} \end{pmatrix},$$

and the probability of developing symptoms (given survival) following infection is the ratio of the transition rate to pre-symptomatic infection to the total infection rate

$$\text{Probability of developing symptoms} = \frac{q_{12}}{q_{12} + q_{16}}.$$

Figure A4: Flow diagram for 6-state Markov model with separate states for asymptomatic and pre-symptomatic infected individuals.

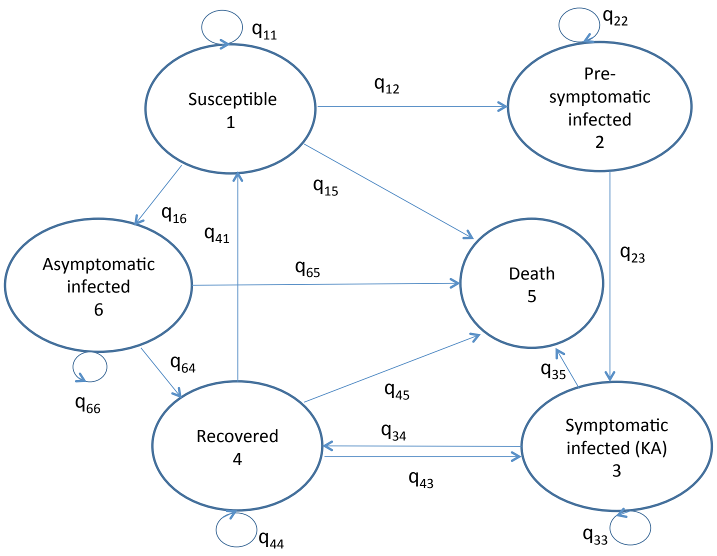

DOI 10.1186/s13071-015-1136-3

The fitted transition intensity matrix for this model is

$$Q_A = \begin{pmatrix} -0.22 & 0.03 & 0 & 0 & 0.005 & 0.18 \\ 0 & -2.70 & 2.70 & 0 & 0 & 0 \\ 0 & 0 & -2.87 & 2.75 & 0.12 & 0 \\ 0.31 & 0 & 0.02 & -0.33 & 0.005 & 0 \\ 0 & 0 & 0 & 0 & 0 & 0 \\ 0 & 0 & 0 & 2.27 & 0.02 & -2.30 \end{pmatrix}.$$

and the estimated proportion of infected individuals that develop KA is 13.8% (95% bootstrap CI 9.7–19.4%). This is very similar to the figure of 14.7% (95% CI 12.6-20.0%) from the 5-state model. The mean times spent in the different disease states are shown in Table A8, and are also very similar to those for the 5-state model (Table A2). Pre-symptomatic individuals appear to progress to KA more quickly on average than asymptomatic individuals recover from infection (in 135 days, 95% CI 109–167 days, compared to 159 days, 95% CI 138–183 days), but there is considerable overlap in the 95% confidence intervals for these estimates, so it is not clear that the difference is significant. The mean duration of KA is shorter for the 6-state model than the 5-state model (127 days, 95% CI 113–143 days, as opposed to 147 days, 95% CI 123–160 days), but the 95% confidence intervals are again overlapping. The 6-state model has a negative log-likelihood of 1717.1 and an Akaike information criterion (AIC) value of 3456.1, which is much lower than for the 5-state model (AIC=3541.1), implying that separating pre-symptomatics and asymptomatics into two states does yield a significant improvement in the fit of the model to the data. However, the similarity in the model estimates suggests that grouping pre-symptomatic and asymptomatic individuals together is a reasonable modelling assumption, and does not significantly bias the results of the model fitting.

Table A8: Mean durations of different disease stages in 6-state Markov model.

Disease state	Mean waiting time (95% CI) (days)
Susceptible	1696 (1494–1925)
Pre-symptomatic	135 (109–167)
KA	127 (113–143)
Recovered/Dormant	1108 (987–1244)
Asymptomatic	159 (138–183)

Author details
[1]School of Life Sciences, University of Warwick, Gibbet Hill Campus, Coventry CV4 7AL , UK. [2]Country Programme Manager - Bangladesh, KalaCORE Programme, Dhaka, Bangladesh. [3]Department of Medical Entomology, National Institute of Preventive and Social Medicine (NIPSOM), Mohakhali, Dhaka, Bangladesh. [4]UCSF School of Medicine, 550 16th Street, San Francisco CA 94158, USA. [5]London School of Hygiene and Tropical Medicine, Keppel Street, London WC1E 7HT, UK.

References

1. Cox D, Miller H. The theory of stochastic processes. New York: Chapman & Hall; 1965.
